# Interstitial lung disease is a risk factor for ischaemic heart disease and myocardial infarction

**DOI:** 10.1136/heartjnl-2019-315511

**Published:** 2020-02-29

**Authors:** Lorna Elise Clarson, Ram Bajpai, Rebecca Whittle, John Belcher, Alyshah Abdul Sultan, Chun Shing Kwok, Victoria Welsh, Mamas Mamas, Christian D Mallen

**Affiliations:** 1 School of Primary, Community and Social Care, Keele University, Keele, UK; 2 Cardiovascular Research Group, Keele University, Stoke-on-Trent, UK

**Keywords:** cardiac risk factors and prevention, coronary artery disease

## Abstract

**Objectives:**

Despite many shared risk factors and pathophysiological pathways, the risk of ischaemic heart disease (IHD) and myocardial infarction (MI) in interstitial lung disease (ILD) remains poorly understood. This lack of data could be preventing patients who may benefit from screening for these cardiovascular diseases from receiving it.

**Methods:**

A population-based cohort study used electronic patient records from the Clinical Practice Research Datalink and linked Hospital Episode Statistics to identify 68 572 patients (11 688 ILD exposed (mean follow-up: 3.8 years); 56 884 unexposed controls (mean follow-up: 4.0 years), with 349 067 person-years of follow-up. ILD-exposed patients (pulmonary sarcoidosis (PS) or idiopathic pulmonary fibrosis (PF)) were matched (by age, sex, registered general practice and available follow-up time) to patients without ILD or IHD/MI. Rates of incident MI and IHD were estimated. HRs were modelled using multivariable Cox proportional hazards regression accounting for potential confounders.

**Results:**

ILD was independently associated with IHD (HR 1.85, 95% CI 1.56 to 2.18) and MI (HR 1.74, 95% CI 1.44 to 2.11). In all disease categories, risk of both IHD and MI peaked between ages 60 and 69 years, except for the risk of MI in PS which was greatest <50 years. Men with PF were at greatest risk of IHD, while women with PF were at greatest risk of MI.

**Conclusions:**

ILD, particularly PF, is independently associated with MI and IHD after adjustment for established cardiovascular risk factors. Our results suggest clinicians should prioritise targeted assessment of cardiovascular risk in patients with ILD, particularly those aged 60–69 years. Further research is needed to understand the impact of such an approach to risk management.

## Background

The interstitial lung diseases (ILDs) are a heterogenous group of diffuse parenchymal lung disorders characterised by fibrotic and/or inflammatory changes to the interstitial lung tissue.^1 2^ The most common of these conditions is idiopathic pulmonary fibrosis (PF), which affects between 14 and 63 per 100 000 population in the USA, and between 1.25 and 23.4 per 100 000 population in Europe.^3^ It is considered progressive and fatal; however, the course of the disease is variable with evidence suggesting comorbidity burden contributes significantly to risk of disease progression and excess mortality.^4 5^ Pulmonary sarcoidosis (PS) has traditionally been considered a more benign condition; however, an increasing trend in rates of mortality and hospitalisation of patients with sarcoidosis has been reported, with comorbid conditions thought to play an important role in this.^6^

Ischaemic heart disease (IHD) is prevalent among patients with ILD with rates of IHD among patients with interstitial pulmonary fibrosis (IPF) reported to be as high as 68%.^4^ IHD is the second most common cause of death in patients with IPF, after IPF itself.^7–9^ The underlying pathogenesis of ILD and IHD share a number of pathophysiological mechanisms including oxidative stress, vascular endothelial injury and release of proinflammatory cytokines associated with a hypercoagulable state and formation of microthrombi.^10 11^ Despite recognition of their coexistence, shared risk factors (RFs), the importance of burden of comorbidities in the disease course of ILD, and the availability of effective primary and secondary IHD prevention measures that could be considered to mitigate any increased risk of IHD in patients with ILD, research examining the risk of incident IHD in patients with ILD are few in number, small in terms of sample size and report lengths of follow-up potentially inadequate to detect the outcomes of interest.^12–15^

This study therefore aimed to investigate the risk of incident IHD and myocardial infarction (MI) in a large cohort of patients with ILD, compared with those without, using linked data from primary and secondary care.

## Methods

### Data source and study population

The Clinical Practice Research Datalink (CPRD) is one of the largest databases of electronic primary care records in the world, covering approximately 7% of the UK population, and is representative of the broader UK population. Practices record patient demographics, consultations, hospitalisations, specialist referrals, prescriptions, test results, immunisations and diagnoses. Clinical information is entered using Read codes, a standard clinical terminology system used in general practice in the UK. Prescriptions are recorded using Multilex (or British National Formulary) codes. The quality of the data is regulated by the Medicines and Healthcare Products Regulatory Authority and only used when it has reached a certain standard of quality. High validity of diagnostic Read codes has been demonstrated within the CPRD.^16 17^ CPRD data can now be linked with Hospital Episode Statistics (HES), which has details of all NHS inpatient care, hospital outpatient visits and emergency hospital attendance in England. Data from private patients treated in NHS hospitals and care delivered by treatment centres funded by the NHS are also included. Demographic data, along with discharge diagnoses and procedures, are recorded using the International Classification of Diseases version 10 and Operation and Procedure Coding Supplement version 4, respectively, and are linked to primary care data by a trusted third-party using NHS number, date of birth and sex. The CPRD-HES linked data covers more than 3% of the total English population and are representative of the general UK population.^18^

ILD exposure was defined as a recorded diagnosis of PS or PF (including idiopathic PF, cryptogenic fibrosing alveolitis and idiopathic fibrosing alveolitis) in their general practice electronic health record (EHR) between 1998 and 2017 using Read codes (available at https://www.keele.ac.uk/mrr). The date associated with the first-ever Read code for the conditions of interest was taken to be the date of diagnosis (‘Index date’). Each incident case was matched to five controls without a diagnosis of either condition on age (±5 years, sex, available follow-up time in CPRD (±3 years) and registered general practice. Unexposed patients were assigned the same index date as their matched ILD-exposed patient and for both, follow-up commenced from that index date. Those with a prior history of IHD (including coronary artery disease (CAD), angina and acute coronary syndrome) or MI, or less than 1 year of follow-up after the index date were excluded. Exposed and unexposed participants were required to be over age 18 years at diagnosis of ILD (or matched index date), have linkage to HES data to identify outcomes of interest, have no history of any of the outcomes of interest prior to the diagnosis of ILD and have 6-month up-to-standard data follow-up in CPRD.

The validity of diagnostic Read codes in CPRD for one of the rarer forms of inflammatory lung disease, cryptogenic fibrosing alveolitis, has been assessed by comparison with hospital documentation and was shown to be high with a positive predictive value of 95%.^19^

### Outcome definition

The primary study outcomes were IHD (including CAD, angina and acute coronary syndrome) and MI identified using medical codes assigned by GPs, or in secondary care HES data (list of Read codes used to identify outcomes available at https://www.keele.ac.uk/mrr). Validity of general practice diagnoses has been reported to be high, with a positive predictive of 85.3% for circulatory diseases,^17^ and 82%–92% for acute MI.^17^

### Covariates

Potential confounders considered were body mass index (BMI), smoking status and alcohol consumption (yes, no, ex or unknown), hypertension, hyperlipidaemia, diabetes, chronic kidney disease, prescription for cardiovascular drugs (defined as at least 30 days supply of an antihypertensive, antiplatelet or lipid-lowering medication), reported family history of cardiovascular disease and socioeconomic status defined based on the area in which the general practice at which the patient was registered is located (quintiles by rank of Indices of Multiple Deprivation).^20^ They were identified closest to the index date from both the primary care EHR and linked HES data.

### Statistical analysis

Absolute rates (ARs) of IHD and MI per 10 000 person-years (PY) and 95% CIs were calculated for cases and controls. Cox proportional hazards regression was used to calculate HRs adjusted for the stated confounding factors. Those with missing BMI data were categorised into a separate ‘missing’ category and included in the analysis, as there is evidence that in this database, BMI data cannot be assumed to be missing completely at random, and therefore may not satisfy the ‘missing at random’ assumption required for multiple imputation.^21^ The proportional hazards assumption was tested using Schoenfeld residuals plot. All statistical analyses were performed using Stata V.15.1. This study was reviewed and approved by the CPRD’s in-house Independent Scientific Advisory Committee (ISAC) reference number: 15_214RA

As we have a matched sample, this introduces a bias that must be accounted for in the analysis stage. Matched subjects will have correlation (greater similarity) in outcomes than two randomly selected subjects. This is because their baseline covariates are more similar, and baseline covariates are related to outcomes. We must therefore account for the lack of independence in outcomes that have been induced by matching. Hence, to account for the matched nature of the sample, we use a robust variance estimator that accounts for the clustering within matched sets.^22^

### Sensitivity analysis

Two sensitivity analyses were performed. First, analyses were repeated removing patients with a coded diagnosis of cardiac sarcoidosis during the time period of observation in order to understand the impact of sarcoidosis affecting the cardiac system on our findings. Second, analyses were repeated introducing number of GP consultations during the period of observation as a variable to investigate the potential impact of surveillance bias.

### Patient and public involvement

Patients were involved in prioritising the research question but, due to the nature of the study using a database of electronic healthcare records, they were not involved further in the design, conduct or reporting of this work.

## Results

### Basic characteristics

Data were analysed for 11 688 ILD-exposed participants, and 56 884 unexposed matched controls, over a total of 349 067 PY. Table 1 presents the characteristics of participants.

**Table 1:** Characteristics of participants

	ILD (n=11 688)	PS (n=4568)	PF (n=7120)	Non-ILD controls (n=56 884)
Mean age (SD)	62.2 (16.8)	48.0 (12.9)	71.4 (11.9)	64.7 (16.3)
Male, n (%)	6291 (53.8)	2313 (50.6)	3978 (55.8)	31 854 (56.0)
Median follow-up, years (IQR)	3.8 (1.8–7.3)	6.1 (2.8–10.5)	3.0 (1.5–5.4)	4.0 (2.0–7.4)
Hypertension, n (%)	3577 (30.6)	752 (16.5)	2825 (39.7)	17 030 (29.9)
Hyperlipidaemia, n (%)	1461 (12.5)	311 (6.8)	1150 (16.2)	6533 (11.5)
Diabetes mellitus, n (%)	1344 (11.5)	345 (7.6)	999 (14.0)	4995 (8.8)
Chronic kidney disease, n (%)	1173 (10.0)	179 (3.9)	994 (14.0)	3998 (7.0)
BMI (kg/m^2^)*				
Normal (≥18.5 ≤24.9)	3430 (29.4)	1256 (27.5)	2174 (30.5)	16 983 (30.0)
Underweight (<18.5)	289 (2.5)	63 (1.4)	226 (3.2)	1166 (2.1)
Overweight (≥25.0 ≤29.9)	4081 (34.9)	1528 (33.5)	2553 (35.9)	17 768 (31.2)
Obese (>30)	2890 (24.7)	1293 (28.3)	1597 (22.4)	10 682 (18.8)
Not recorded	998 (8.5)	428 (9.4)	570 (8.0)	10 285 (18.1)
Alcohol consumption				
No	2286 (19.6)	836 (18.3)	1450 (20.4)	9284 (16.3)
Yes	8058 (68.9)	3221 (70.5)	4837 (67.9)	36 533 (64.2)
Ex	356 (3.05)	89 (1.9)	267 (3.8)	1395 (2.5)
Not recorded	988 (8.45)	422 (9.2)	566 (7.9)	9672 (17.0)
Smoking history				
No	5867 (50.2)	3005 (65.8)	2862 (40.2)	27 406 (48.2)
Yes	1453 (12.4)	518 (11.3)	935 (13.1)	9406 (16.5)
Ex	4188 (35.8)	986 (21.6)	3202 (45.0)	14 579 (25.6)
Not recorded	180 (1.5)	59 (1.3)	121 (1.7)	5493 (9.7)
Family history of CVD	2410 (20.6)	920 (20.1)	1490 (20.9)	10 545 (18.5)
Socioeconomic status (quintiles of Index of Multiple Deprivation)				
1 (least deprived)	1638 (24.7)	702 (15.4)	936 (13.1)	7548 (13.3)
2	1535 (23.2)	611 (13.4)	924 (13.0)	7639 (13.4)
3	1302 (19.7)	493 (10.8)	809 (11.4)	6689 (11.8)
4	1203 (18.2)	482 (10.6)	721 (10.1)	5656 (9.9)
5 (most deprived)	942 (14.2)	386 (8.5)	556 (7.8)	4475 (7.9)
Exposure to cardiovascular drugs				
Antihypertensive	3494 (29.9)	717 (15.7)	2777 (39.0)	13 594 (23.9)
Antiplatelet	768 (6.6)	101 (2.2)	667 (9.4)	3160 (5.6)
Lipid lowering	1071 (9.2)	235 (5.1)	836 (11.7)	4449 (7.8)

*WHO Classification of Obesity According to BMI.

BMI, body mass index; CVD, cardiovascular disease; ILD, inflammatory lung disease; PF, pulmonary fibrosis; PS, pulmonary sarcoidosis.

Patients with PS were younger, had a lower burden of cardiovascular RF and longer median duration of follow-up than either patients with PF or controls.

### Myocardial infarction

The AR of MI was highest in patients with PF (AR 72.57 (95% CI 62.95 to 83.65) per 10 000 PY). Rates of MI were lower in those exposed to PS than unexposed patients (16.01 (95% CI 12.17 to 21.07) vs 32.26 (95% CI 30.26 to 34.39) per 10 000 PY) (table 2).

**Table 2:** Myocardial infarction incidence rates per 10 000 person-year of follow-up by exposure status

Variables	Unexposed			ILD combined			PS			PF		
	N	PY	Rate* (95% CI)	N	PY	Rate* (95% CI)	N	PY	Rate* (95% CI)	N	PY	Rate* (95% CI)
Overall	56 884	291 038	32.26 (30.26 to 34.39)	11 688	58 029	41.53 (36.60 to 47.12)	4568	31 846	16.01 (12.17 to 21.07)	7120	26 183	72.57 (62.95 to 83.65)
Sex												
Male	31 854	151 469	40.27 (37.20 to 43.60)	6291	29 195	54.12 (46.31 to 63.25)	2313	15 473	20.68 (14.63 to 29.24)	3978	13 722	91.83 (77.11 to 109.34)
Female	25 030	139 569	23.57 (21.16 to 26.26)	5397	28 835	28.78 (23.21 to 35.69)	2255	16 373	11.60 (7.40 to 18.19)	3142	12 462	51.36 (40.20 to 65.62)
Age (years)												
<50	12 272	94 517	4.97 (3.74 to 6.62)	3075	22 576	10.63 (7.13 to 15.86)	2691	20 207	7.92 (4.85 to 12.92)	384	2369	33.77 (16.89 to 67.51)
50–59	7460	48 505	16.29 (13.06 to 20.31)	1759	10 830	27.70 (19.37 to 39.62)	1047	7091	21.15 (12.75 to 35.09)	712	3740	40.11 (24.18 to 66.53)
60–69	11 321	57.911	30.39 (26.22 to 35.23)	2308	10 643	63.89 (50.38 to 81.04)	568	3376	29.62 (15.94 to 55.04)	1740	7267	79.81 (61.71 to 103.24)
70–79	15 392	60 347	64.63 (58.52 to 71.37)	2759	9443	80.48 (64.27 to 100.77)	210	1004	89.60 (46.62 to 172.21)	2549	8439	79.39 (62.49 to 100.87)
>80	10 438	29 757	83.01 (73.27 to 94.03)	1786	4536	94.79 (70.30 to 127.81)	52	168	59.67 (8.41 to 423.59)	1735	4369	96.13 (71.05 to 130.09)

*Rate per 10 000 person-years.

ILD, inflammatory lung disease; N, number; PF, pulmonary fibrosis; PY, person-years.

Risk of MI compared with unexposed patients was similar between the ILD combined group patients and PF group (HR 1.53 (95% CI 1.33 to 1.77 vs HR 1.58 (95%CI 1.35 to 1.85)). The PS group was not at increased risk of MI. While for men, risks of MI were similar across each of the disease categories, women with PF were at greatest risk of MI and greater than their male counterparts. In ILD and PF risk of MI peaked between ages 60 years and 69 years; however, in PS the youngest cohort of patients (<50 years) were at greatest risk.

### Ischaemic heart disease

The AR of IHD was also highest in patients with PF (102.61 (95% CI 91.06 to 115.64) per 10 000 PY). Rates of IHD were also lower in those exposed to PS than unexposed patients (20.09 (95% CI 15.73 to 25.67) vs 39.71 (95% CI 37.49 to 42.07) per 10 000 PY) (table 3).

**Table 3:** Ischaemic heart disease incidence rates per 10 000 person-year (PY) of follow-up by exposure status

Variables	Unexposed			ILD combined				PS			PF	
	N	PY	Rate* (95% CI)	N	PY	Rate* (95% CI)	N	PY	Rate* (95% CI)	N	PY	Rate* (95% CI)
Overall	56 884	291 098	39.71 (37.49 to 42.07)	11 688	58 064	57.35 (51.51 to 63.85)	4568	31 849	20.09 (15.73 to 25.67)	7120	26 215	102.61 (91.06 to 115.64)
Sex												
Male	31 854	151 493	52.74 (49.21 to 56.53)	6291	29 209	78.06 (68.56 to 88.88)	2313	15 471	20.68 (14.63 to 29.25)	3978	13 737	142.68 (124.03 to 164.12)
Female	25 030	139 605	25.57 (23.05 to 28.37)	5397	28 855	36.39 (30.05 to 44.06)	2255	16 377	19.54 (13.82 to 27.67)	3142	12 477	58.51 (46.51 to 73.59)
Age (years)												
<50	12 272	94 514	6.35 (4.93 to 8.18)	3075	22 579	9.74 (6.42 to 14.80)	2691	20 210	7.42 (4.47 to 12.31)	384	2369	29.55 (14.09 to 61.98)
50–59	7460	48 513	24.94 (20.87 to 29.81)	1759	10 841	37.82 (27.85 to 51.36)	1047	7098	29.59 (19.29 to 45.38)	712	3740	53.43 (34.47 to 82.82)
60–69	11 321	57 927	50.06 (44.62 to 56.17)	2308	10 638	98.70 (81.52 to 119.51)	568	3367	62.37 (40.67 to 95.66)	1740	7271	115.52 (93.28 to 143.07)
70–79	15 392	60 369	79.68 (72.86 to 87.12)	2759	9460	130.03 (108.96 to 155.16)	210	1006	59.61 (26.78 to 132.69)	2549	8453	138.41 (115.47 to 165.91)
>80	10 438	29 774	68.52 (59.73 to 78.59)	1786	4546	92.39 (68.28 to 125.02)	52	168	59.56 (8.39 to 422.79	1735	4377	93.65 (68.96 to 127.19)

ILD, interstitial lung disease; PF, pulmonary fibrosis; PS, pulmonary sarcoidosis.

ILD exposure was associated with a 59% increased risk of IHD compared with unexposed patients (HR 1.58, 95% CI 1.37 to 1.83). Patients with PF were at greatest risk (HR 1.78, 95% CI 1.51 to 2.09), while patients with PS were not at any increased risk of IHD.

**Figure 1:**
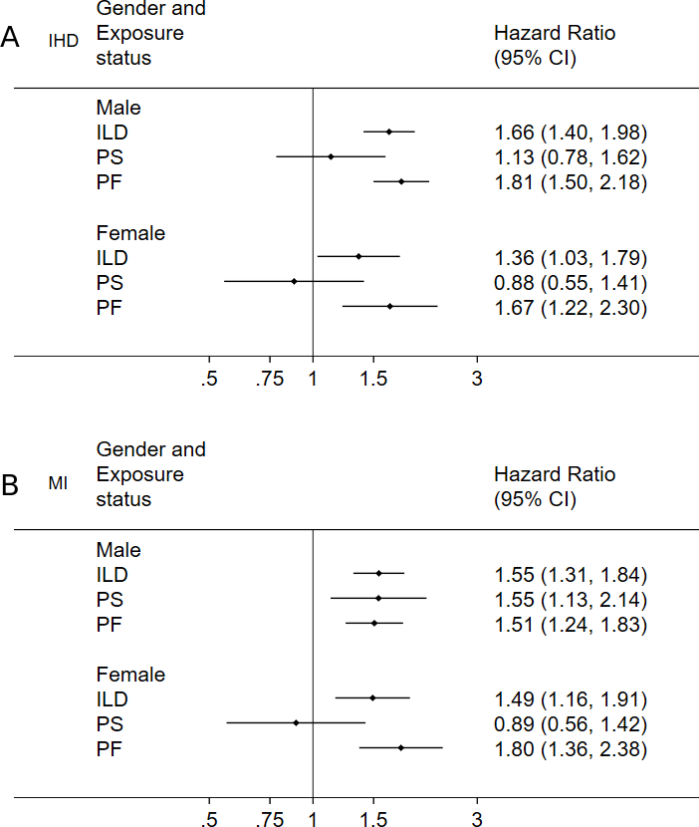
Risk of IHD (A) and myocardial infarction (B) by gender and exposure status. IHD, ischaemic heart disease; ILD, interstitial lung disease; PF, pulmonary fibrosis; PS, pulmonary sarcoidosis.

**Figure 2:**
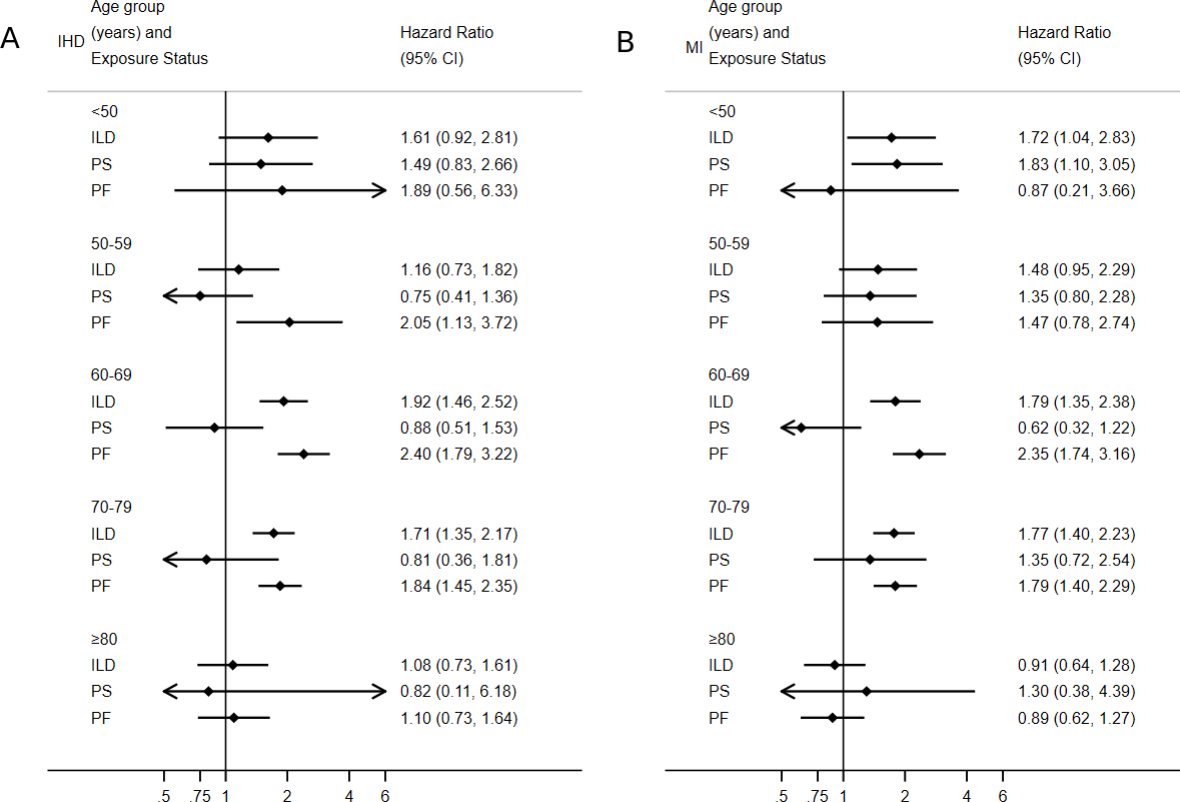
Risk of IHD (A) and myocardial infarction (B) by age and exposure status. IHD, ischaemic heart disease; ILD, interstitial lung disease; PF, pulmonary fibrosis; PS, pulmonary sarcoidosis.

Risk of IHD was greatest in men, while risk of MI was greatest in women (table 4; figure 1). Risk of both IHD and MI peaked in the 60–69 age group, with the exception of PS where risk of MI was greatest in patients <50 years (table 4; figure 2).

**Table 4:** Risk of ischaemic heart disease (IHD) and myocardial infarction (MI) by exposure status

	IHD		MI	
	Male	Female	Male	Female
	HR (95% CI)	HR (95% CI)	HR (95% CI)	HR (95% CI)
ILD	1.66 (1.40 to 1.98)	1.36 (1.03 to 1.79)	1.55 (1.31 to 1.84)	1.49 (1.16 to 1.91)
PS	1.13 (0.78 to 1.63)	0.88 (0.55 to 1.41)	1.55 (1.13 to 2.14)	0.89 (0.56 to 1.42)
PF	1.81 (1.50 to 2.18)	1.67 (1.22 to 2.30)	1.51 (1.24 to 1.83)	1.80 (1.36 to 2.38)

Age (years)	IHD (HR (95% CI))			MI (HR (95% CI))		
	ILD	PS	PF	ILD	PS	PF
<50	1.61 (0.92 to 2.81)	1.49 (0.83 to 2.66)	1.89 (0.56 to 6.33)	1.72 (1.04 to 2.83)	1.83 (1.10 to 3.05)	0.87 (0.21 to 3.66)
50–59	1.16 (0.73 to 1.82)	0.75 (0.41 to 1.36)	2.05 (1.13 to 3.72)	1.48 (0.95 to 2.29)	1.35 (0.80 to 2.28)	1.47 (0.78 to 2.74)
60–69	1.92 (1.46 to 2.52)	0.88 (0.51 to 1.53)	2.40 (1.79 to 3.22)	1.79 (1.35 to 2.38)	0.62 (0.32 to 1.22)	2.35 (1.74 to 3.16)
70–79	1.71 (1.35 to 2.17)	0.81 (0.36 to 1.81)	1.84 (1.45 to 2.35)	1.77 (1.40 to 2.23)	1.35 (0.72 to 2.54)	1.79 (1.40 to 2.29)
>80	1.08 (0.73 to 1.61)	0.82 (0.11 to 6.18)	1.10 (0.73 to 1.64)	0.91 (0.64 to 1.28)	1.30 (0.38 to 4.39)	0.89 (0.62 to 1.27)

All analyses adjusted for CKD, HTN, DM, HLD, BMI, exposure to smoking and alcohol, IMD, family history of cardiovascular disease and exposure to antihypertensive, antiplatelet and lipid-lowering drugs.

BMI, body mass index; CKD, chronic kidney disease; DM, diabetes mellitus; HLD, hyperlipidaemia; HR, adjusted HR; HTN, hypertension; ILD, inflammatory lung disease; IMD, Index of Multiple Deprivation; PF, pulmonary fibrosis; PS, pulmonary sarcoidosis.

Results of the sensitivity analysis excluding patients with cardiac sarcoidosis did not reveal any material change in the findings (online supplementary table S1). The associations identified in the main analysis were strengthened in the sensitivity analysis adjusting for number of GP consultations (online supplementary table S2).

10.1136/heartjnl-2019-315511.supp1 Supplementary data

10.1136/heartjnl-2019-315511.supp2 Supplementary data

## Conclusion

### Main findings

Both men and women with ILD were at increased risk of IHD and MI compared with unexposed controls. PF patients have a 2–3 fold greater incidence of acute myocardial infarction (AMI) and IHD in younger age groups compared with PS and unexposed controls. Women with PF were at greatest risk of MI (approximately 80% excess risk), while men with PF were at greatest risk of IHD (also approximately 80% excess risk). Women with PS were not at any increased risk of IHD or MI. Men with ILD overall experienced a slightly higher risk of IHD and MI to that in women. In all disease categories, risk of both IHD and MI peaked between ages 60 years and 69 years, except for the risk of MI in PS which was greatest in the youngest age group (<50 years).

### Strengths of this study

This study is the first to report the risk of MI in ILD and to report risks of IHD and MI by age and gender, identifying women with PF and patients <50 years with PS to be at greatest risk of MI. This study is also the first to adjust for important factors such as deprivation. The use of GP electronic medical records that are kept contemporaneously limits the risk of recall or observer bias and allows a long duration of patient follow-up. In addition, our study used linked secondary care data, which have been shown to ascertain outcomes more robustly.^23^

However, the findings of this study should be interpreted with caution due to the risk of misclassification resulting from the use of EHRs, although the validity of certain forms of inflammatory lung disease, including IPF diagnosis has previously been examined in such databases and was found to be high.^20^ The risk of surveillance bias should also be considered as it is possible that patients with ILD see their GP more frequently or are investigated in more detail than those in the general population that may lead to increased opportunities for detection of disease, although the findings of our sensitivity analysis would refute this. Finally, while we adjust for the presence of RFs such as hypertension, we do not adjust for their severity, and there may be a differential impact on risk across a range of blood pressure values.

### Interpretation in the context of other studies

While other studies do not report risk of IHD by age and sex, our overall findings are in line with those of previously published studies reporting an increased risk of IHD in patients with ILD.^9 12–16 24–26^ Two smaller studies that examined this association in an alternative database of UK primary care EHRs (The Health Improvement Network) reported an increased incidence of CAD or angina associated with IPF. After adjustment for age, sex and smoking status, Hubbard *et al* 12 reported a rate ratio of incident CAD in 920 patients with IPF compared with 3593 controls of 3.39 (95% CI 2.02 to 4.87), which is slightly higher than the rate ratio in this study of 2.39 (95% CI 2.11 to 2.70) but may reflect the wider range of cardiovascular RFs adjusted for in this study. Dalleywater *et al* reported a rate ratio of IHD 2.32 (95% CI 1.85 to 2.93) in 3211 participants with IPF compared with 12 307 controls, after adjustment for hypertension, hypercholesterolaemia, diabetes, smoking status and BMI, similar to the findings of this study.^15^ A further study of 406 Korean patients with IPF reported that the incidence of CAD was higher (6.8%) compared with controls (2.8%) with a relative risk of 1.92 (95% CI 1.08 to 3.43), also similar in magnitude to our findings.^16^ Increased prevalence of angiographically determined CAD in patients with PF has also been reported, with an OR of 2.18 for the presence of angiographic CAD in patients with fibrotic versus non-fibrotic lung disease.^24^ Significantly more CAD was reported in 49 patients with lung fibrosis (28.6%) compared with 51 with emphysema (9.8%, p=0.02 for difference) despite there being significantly more smokers in the emphysema group (98% vs 31%),^25^ and prevalence of CAD was higher in pretransplant IPF patients (65.8%) than pretransplant chronic obstructive pulmonary disease patients (46.1%, p=0.03 for difference) independent of CAD RFs. IPF patients with severe CAD also had worse outcomes than controls with a similar disease burden in this study.^26^

In common with other studies, there was an increased prevalence of established cardiovascular RFs in our ILD and PF cohorts,^15^ and while we adjusted for the presence of these RFs, we could not adjust for their severity. It is therefore possible that this association reflects either less well controlled cardiovascular RFs in patients with ILD/PF or the presence of unmeasured novel cardiovascular RFs.

It has also been suggested that the presence of a serious lung disease might distract medical attention from routine cardiovascular care and that either primary and secondary prevention interventions are neglected, or symptoms that might be suggestive of cardiovascular disease are falsely attributed to the known respiratory condition.^12^

Several pathophysiological mechanisms may account for the association between ILD and CAD. Autoantibodies such as antinuclear antibody and antiendothelial cell antibodies are known to circulate in higher concentrations in patients with IPF than the general population, and deposition of such autoantibodies in the endothelial cells of lung tissue may stimulate the release of proinflammatory cytokines and monocyte recruitment and activation.^10^ In addition, oxidative stress may play a role.^11^ This triggering of the inflammatory cascade has been hypothesised to cause endothelial injury or necrosis and is also associated with a hypercoagulable state, the formation of microthrombi and the activation of fibroblasts in common with mechanisms that have been implicated in IHD and MI.^10 27 28^ Patients with IPF have been reported to have an increased tendency to thromboembolic disease and to have higher factor VIII levels than the general population, with elevated factor VIII levels also associated with an increased risk of CAD.^29^

In common with other systemic inflammatory disorders, this study demonstrates that the presence of PF provides independent information to the incident risk of MI/IHD on top of classical cardiovascular RFs. This has led to both rheumatoid arthritis (RA) and systemic lupus erythematosus (SLE) being included in cardiovascular risk stratification tools.^30^ The increase in risk of AMI/IHD associated with PF/ILD in this study exceeds that attributed to RA within the preferred model of newest iteration of the QRISK tool (HR men 1.23 (95% CI 1.19 to 1.28); women 1.24 (95% CI 1.20 to 1.27)) and in younger age groups also exceeds that attributed to SLE (HR men 1.55 (95% CI 1.15 to 2.10); women 2.14 (95% CI 1.78 to 2.56)).^30^ It could therefore be argued that ILD should be considered in future iterations of cardiovascular risk stratification tools.

### Conclusion

Our results enable clinicians to identify which cohorts of ILD patients are most at risk of IHD and MI and target their assessment of cardiovascular risk appropriately. Our results also suggest that a more aggressive approach to primary prevention may be warranted in certain groups of ILD patients, for example, those <50 years old with PS. Further research is needed to understand the impact of such a stratified approach to risk management.

**Key messages**

**What is already known on this subject?**

- In common with other inflammatory diseases, such as rheumatoid arthritis (RA) and systemic lupus erythematosus (SLE), interstitial lung disease (ILD) and ischaemic heart disease (IHD) share a number of pathophysiological mechanisms and common risk factors. However, unlike these other diseases, the risk of IHD in patients with ILD is poorly understood. This lack of data could be preventing patients with ILD who may benefit from screening for these cardiovascular diseases from receiving it, as patients with RA and SLE have.

**What might this study add?**

- This study is the first to report the risk of myocardial infarction (MI) in ILD and to report risks of IHD and MI by age and gender, identifying women with pulmonary fibrosis (PF) and patients <50 years with pulmonary sarcoidosis to be at greatest risk of MI. This study is also the first to adjust for important factors such as deprivation.
- This study demonstrates that the presence of PF provides independent information to the incident risk of MI/IHD on top of classical cardiovascular RFs.

**How might this impact on clinical practice?**

- The increase in risk of AMI/IHD associated with PF/ILD in this study exceeds that attributed to RA within the preferred model of newest iteration of the QRISK tool and in younger age groups also exceeds that attributed to SLE. It could therefore be argued that in common with recommendations for RA/SLE, clinicians should screen patients with ILD for IHD, and furthermore, ILD should be considered in future iterations of cardiovascular risk stratification tools.
