# Supplementary material for: Interstitial lung disease is a risk factor for ischaemic heart disease and myocardial infarction
Source: Heart. 2020 Feb 29;106(12):916–22. doi: 10.1136/heartjnl-2019-315511 (PMC7282497; doi:10.1136/heartjnl-2019-315511)
Supplement: Supplementary data [file heartjnl-2019-315511supp001.pdf]

Table S1: Risk of Ischaemic Heart Disease and Myocardial Infarction by Exposure Status Excluding Cardiac Sarcoidosis Cases (n=31)

	**IHD**		**MI**	
	**Male**	**Female**	**Male**	**Female**
	**HR (95%CI)**	**HR (95%CI)**	**HR (95%CI)**	**HR (95%CI)**
**ILD**	**1.67 (1.40-1.98)**	**1.36 (1.03-1.79)**	**1.54 (1.30-1.83)**	**1.49 (1.16-1.91)**
**PS**	1.13 (0.79-1.63)	0.88 (0.55-1.41)	**1.50 (1.08-2.07)**	0.89 (0.56-1.42)
**PF**	**1.81 (1.50-2.18)**	**1.67 (1.22-2.30)**	**1.51 (1.25-1.83)**	**1.80 (1.36-2.38)**

Age

	**IHD**			**MI**		
	**ILD**	**PS**	**PF**	**ILD**	**PS**	**PF**
**<50**	1.62 (0.93-2.83)	1.50 (0.84-2.68)	1.88 (0.56-6.32)	1.63 (0.98-2.72)	**1.72 (1.02-2.91)**	0.92 (0.22-3.89)
**50-59**	1.16 (0.74-1.83)	0.75 (0.42-1.36)	**2.05 (1.13-3.71)**	1.48 (0.95-2.29)	1.35 (0.80-2.28)	1.47 (0.79-2.74)
**60-69**	**1.92 (1.46-2.52)**	0.88 (0.51-1.53)	**2.40 (1.79-3.22)**	**1.80 (1.35-2.38)**	0.62 (0.32-1.23)	**2.35 (1.74-3.16)**
**70-79**	**1.71 (1.35-2.17)**	0.81 (0.36-1.82)	**1.84 (1.45-2.35)**	**1.77 (1.40-2.23)**	1.35 (0.72-2.55)	**1.79 (1.41-2.29)**
**>80**	1.08 (0.73-1.61)	0.83 (0.11-6.18)	1.10 (0.73-1.64)	0.91 (0.64-1.28)	1.30 (0.39-4.39)	0.88 (0.62-1.27)

All analyses adjusted for CKD, HTN, DM, HLD, BMI, exposure to smoking and alcohol, IMD, family history of cardiovascular disease and exposure to anti-hypertensive, anti-platelet and lipid-lowering drugs

CI=Confidence Interval; CKD=Chronic Kidney Disease; DM=Diabetes Mellitus; HLD=Hyperlipidaemia; HR= adjusted Hazard Ratio; HTN=Hypertension; IHD=Ischaemic Heart Disease; ILD=Inflammatory Lung Disease; IMD=Index of Multiple Deprivation; MI=Myocardial Infarction; PF=Pulmonary Fibrosis; PS=Pulmonary Sarcoidosis
