# Supplementary material for: Interstitial lung disease is a risk factor for ischaemic heart disease and myocardial infarction
Source: Heart. 2020 Feb 29;106(12):916–22. doi: 10.1136/heartjnl-2019-315511 (PMC7282497; doi:10.1136/heartjnl-2019-315511)
Supplement: Supplementary data [file heartjnl-2019-315511supp002.pdf]

Table S2: Risk of Ischaemic Heart Disease and Myocardial Infarction by Exposure Status Adjusted for Number of GP Consultations

	IHD		MI	
	Male	Female	Male	Female
	HR (95%CI)	HR (95%CI)	HR (95%CI)	HR (95%CI)
**ILD**	**1.95 (1.64-2.32)**	**1.58 (1.19-2.08)**	**1.87 (1.57-2.22)**	**1.72 (1.34-2.22)**
**PS**	1.40 (0.97-2.01)	0.97 (0.61-1.55)	**2.00 (1.45-2.75)**	0.95 (0.59-1.51)
**PF**	**2.06 (1.71-2.48)**	**1.97 (1.43-2.72)**	**1.75 (1.44-2.12)**	**2.16 (1.63-2.86)**

**Age**	IHD			MI		
	ILD	PS	PF	ILD	PS	PF
**<50**	1.73 (0.98-3.06)	1.57 (0.87-2.84)	2.10 (0.61-7.29)	**1.84 (1.11-3.07)**	**1.94 (1.15-3.28)**	0.92 (0.21-4.06)
**50-59**	1.42 (0.90-2.28)	0.91 (0.50-1.67)	**2.39 (1.31-4.36)**	**1.96 (1.25-3.06)**	**1.90 (1.12-3.24)**	1.64 (0.86-3.10)
**60-69**	**2.17 (1.65-2.86)**	0.96 (0.56-1.67)	**2.74 (2.04-3.68)**	**2.10 (1.58-2.80)**	0.69 (0.35-1.35)	**2.80 (2.07-3.78)**
**70-79**	**1.94 (1.53-2.46)**	0.86 (0.38-1.94)	**2.10 (1.64-2.68)**	**2.03 (1.61-2.57)**	1.46 (0.78-2.75)	**2.08 (1.63-2.66)**
**>80**	1.30 (0.87-1.95)	0.82 (0.10-6.69)	1.33 (0.88-1.99)	1.09 (0.77-1.55)	1.18 (0.33-4.28)	1.09 (0.76-1.56)

All analyses adjusted for CKD, HTN, DM, HLD, BMI, exposure to smoking and alcohol, IMD, family history of cardiovascular disease and exposure to anti-hypertensive, anti-platelet, lipid-lowering drugs and no of GP consultations

CI=Confidence Interval; CKD=Chronic Kidney Disease; DM=Diabetes Mellitus; HLD=Hyperlipidaemia; HR= adjusted Hazard Ratio; HTN=Hypertension; IHD=Ischaemic Heart Disease; ILD=Inflammatory Lung Disease; IMD=Index of Multiple Deprivation; MI=Myocardial Infarction; PF=Pulmonary Fibrosis; PS=Pulmonary Sarcoidosis
